# Brain-derived Neurotropic factor (BDNF) mediates the protective effect of *Cucurbita pepo* L. on salivary glands of rats exposed to chronic stress evident by structural, biochemical and molecular study

**DOI:** 10.1590/1678-7757-2020-1080

**Published:** 2021-10-04

**Authors:** Hailah M. ALMOHAIMEED, Emad A. ALBADAWI, Zuhair M. MOHAMMEDSALEH, Hadel M. ALGHABBAN, Hanan S. SELEEM, Osama I. RAMADAN, Nasra N. AYUOB

**Affiliations:** 1 Princess Nourah bint Abdulrahman University College of Medicine Department of Basic Science Riyadh Saudi Arabia Princess Nourah bint Abdulrahman University (PNU), College of Medicine, Department of Basic Science, Riyadh, Saudi Arabia.; 2 Taibah University College of Medicine Department of Anatomy Saudi Arabia Taibah University, College of Medicine, Department of Anatomy, Kingdom of Saudi Arabia.; 3 University of Tabuk Faculty of Applied Medical Sciences Department of Medical Laboratory Technology Tabuk Saudi Arabia University of Tabuk, Faculty of Applied Medical Sciences, Department of Medical Laboratory Technology, Tabuk 71491, Saudi Arabia.; 4 University of Taibah Faculty of Applied Medical Sciences Department of Medical Laboratory Technology Saudi Arabia University of Taibah, Faculty of Applied Medical Sciences, Department of Medical Laboratory Technology, Saudi Arabia .; 5 Menoufia University Faculty of Medicine Department of Histology Shebin ElKoumMenofia Egypt Menoufia University, Faculty of Medicine, Department of Histology, Shebin ElKoum, Menofia, Egypt.; 6 Qassim University Unaizah College of Medicine and Medical Sciences Department of Basic Medical Sciences Saudi Arabia Qassim University, Unaizah College of Medicine and Medical Sciences, Department of Basic Medical Sciences, Saudi Arabia.; 7 Al Azhar University Damietta Faculty of Medicine Histology Department Cairo Egypt Al Azhar University, Damietta Faculty of Medicine, Histology Department, Cairo, Egypt.; 8 Damietta University Faculty of Medicine Department of Medical Histology Damietta Egypt Damietta University, Faculty of Medicine, Department of Medical Histology, Damietta, Egypt.

**Keywords:** Chronic stress, Submandibular gland, Parotid gland, Pumpkin, Fluoxetine, Depression, BDNF

## Abstract

Acute and chronic stresses affect the salivary glands, representing the source of plasma BDNF during stressful conditions. Pumpkin is a medicinal plant with an evident antioxidant, anti-inflammatory and potential antidepressant effects.

**Objective:**

To assess the structural and biochemical effects induced by exposure to chronic unpredictable mild stress (CUMS) on salivary glands of albino rats, and to evaluate the role of pumpkin extract (Pump) in ameliorating this effect.

**Methodology:**

Four groups (n=10 each) of male albino rats were included in this study: the control, CUMS, Fluoxetine-treated and Pump-treated. The corticosterone, the pro-inflammatory cytokines, tumor necrosis factor-alpha (TNF-α) and interleukin-6 (IL-6), and the oxidant/antioxidant profile were all assessed in the serum. The level of BDNF mRNA was measured in the salivary glands using qRT-PCR. Histopathological changes of the salivary glands were also assessed.

**Results:**

The depressive-like status was confirmed behaviorally and biochemically. Exposure to CUMS significantly up-regulated (p<0.001) the level of serum corticosterone. CUMS induced degenerative changes in the secretory and ductal elements of the salivary glands evident by increased apoptosis. Both Fluoxetine and Pumpkin significantly up-regulated (p<0.001) BDNF expression in the salivary glands and ameliorated the CUMS-induced histopathological and biochemical alterations in the salivary glands. Pumpkin significantly (p<0.001) increased the serum levels of antioxidant enzymes SOD, GPX and CAT, and reduced the serum levels of the pro-inflammatory cytokines TNF-α, IL-6.

**Conclusion:**

Pumpkin ameliorates the depressive-like status induced in rats following exposure to chronic stress through exerting a promising anti-inflammatory, antioxidant and anti-depressant-like effects. The pumpkin, subsequently, improved stress-induced structural changes in the salivary glands that might be due to up-regulation of BDNF expression in the glands.

## Introduction

Stress is a critical risk factor for numerous neuropsychiatric disorders, including depression and schizophrenia. Moreover, exposure to chronic stress results in neurochemical alterations and depressive behavior.^1^ The World Health Organization (WHO) expected that, in 2020, depression can be the second important disability cause in the world and, in 2030, the major depressive disorders will represent the highest among of healthcare spending.^2^ However, the precise mechanism underlying depression is not fully elucidated and the results obtained from the animal models are not consistent with those of clinical trials.^3^ The model of chronic unpredictable mild stress (CUMS) in rodents has been thought to be the suitable model for discovering the pathophysiology of depression due to its high face, structural, and predictive validity.^4^

Salivary glands are sensitive to stress, and their secretion include brain-derived neurotropic factor (BDNF), which is produced by the salivary glands and amygdala in rats in response to acute stress.^1^ On the other hand, chronic stress reduces BDNF expression in amygdala, hippocampus, and cingulate cortex. This is the reason why several studies on stress-related disorders have focused on it as a biomarker of stress.^1,5^ High level of BDNF is a crucial neuroprotective response under acute immobilization-associated stressful conditions. Furthermore, BDNF crosses the blood brain barrier and plays a vital role in homeostasis under stressful conditions.^6^ However, the relationship between chronic unpredictable stress and salivary compounds remains unclear.

Inflammation was described to be an underlining mechanism in the development of depression induced by chronic stress.^7^ Moreover, previous studies postulated that depression is an oxidative and inflammatory disorder.^8^ Therefore, we assess the inflammatory cytokines tumor necrosis factor-alpha (TNF-α) and interleuin-6 (IL-6) in this study. Fluoxetine (FLU), a classic antidepressant belonging to the selective serotonin reuptake blockers, was used for pharmacological validation of new therapies and drugs.^9^ It also induces its effects on rats exposed to CUMS through reduction of neuroinflammation in prefrontal cortex.^10^

Herbal medicine is used individually or in combination with pharmacological drugs in treatment of different medical disorders. Pumpkin (*Cucurbita pepo L.; Cucurbitaceae*) is a popular plant with many medical properties such as anti-diabetic, anti-oxidants, anti-inflammatory, anti-carcinogens and phytochemical, among others.^11^ Forced-swimming and tail-suspension tests were used to study the antidepressant-like effect of pumpkin seed extract compared to imipramine. Although the extracts showed a significant anti-depressive-like effect, the mechanism of this effect was not clear.^12^ Furthermore, pumpkin seeds are defined among antidepressant food with an score of 47%.^13^ Pumpkin (*Cucurbita moschata*) was has a promising anti-fatigue and an ergogenic activity as it increased the maximal swimming time of in mice a dose-dependent manner.^14^ In a recent study, methanolic extract of *Cucurbita pepo* seeds could inhibit haloperidol-induced motor dysfunction and anxiety and recover locomotor activity.^15^ This study was conducted to assess the structural and biochemical effect induced by exposure to CUMS on the of albino rats’ salivary glands, and to evaluate the role of pumpkin extract in ameliorating this effect. Thus, as the primary outcome of this study, we hypothesized that pumpkin extract ameliorates the chronic stress-induced structural changes in the salivary glands of rats.

## Methodology

### Preparation of pumpkin extract (PE)

Fresh pumpkin *(Cucurbita pepo* L.) was purchased from the local market at Jeddah, Saudi Arabia and was identified by a specialist at the Botany department, Faculty of Science, King Abdulaziz University (KAU). Pumpkin extract was obtained according to the method previously described.^14^ The fruits had their seeds and skin removed, and then were cut with the aid of a slicer. Next, the fruits were dried by using the lyophilize machine freeze-drier (FD5508; ILShinBase Co., Ltd., Korea). Finally, they were crushed by grinding with electrical machine. The powder was passed through a 40-mesh sieve to get the fine powder, which was stored in an airtight container.

The dried powder (50g) of pumpkin was used to prepare the ethanolic extract as was previously described.^16^ The yield of extraction was 41%. PE was stored in a suitable container till use after being dissolved in distilled water at a dose of 100 mg/kg and administered by gavage once daily for two weeks.^14^

### Analysis of the PE

The components of PE were identified using Trace Gas Chromatography and Mass Spectrometer (GC–MS) (Thermo Scientific, Austin, TX, USA) with a direct capillary column TR–5MS (30 m 0.25 µm 9 0.25 µm film thickness.

### Experiment design

The protocol of this study was reviewed and permitted by the Institutional Review Board of Faculty of Medicine, King Abdulaziz University (Code: R.19.12.696). A total of 40 male albino rats weighting 30 g to 40 g and aged 21-28 days, purchased from animal house at King Fahed Medical Research Center, were used. The male gender was selected to nullify the effect of gender as an effect modifier. No other confounders existed in this study. Rats were left to acclimatize in the laboratory conditions under the standard laboratory condition. Each 5 rats were housed in plastic cages in an air-conditioned room at 22±1°C and feed with the standard animal food and water *ad libitum*. At the time of starting the experiment they were weighted 150 to 200 g and aged from 2 to 3 months. The rats were randomly distributed, using simple random technique, into control and experimental groups. The control group included 10 rats that were not exposed to stress. The experimental group included 30 rats that were subjected to CUMS procedure through exposure to diverse types of stressors at different times during the day for four weeks, to prevent habituation to the stressors. The latter included tilting the cage at 30 degrees, placing rats in an empty cage with water at the bottom, with wet sawdust or with in soiled cages of other mice, as well as restrain stress and reversal of the light/dark cycle. The rats were exposed for each stressor for 4 hours at different points during the day to prevent habituation. The CUMS procedure was fully described in previous works.^17^ These 30 rats were then divided into three groups (n=10): untreated (CUMS), FLU-treated group (CUMS+FLU) and Pump-treated (CUMS+Pump) groups. Fluoxetine (Dar Al Dawa Pharmaceuticals Co., Ltd., Amman, Jordan), was dissolved in 0.03% sodium carboxymethyl cellulose (CMC-Na), administered by gavage (20 mg/kg) once daily for two weeks.^18^ After two weeks the behavioral tests were performed and the sampling process was started.

### Assessment of behavioral changes

To confirm the effect of CUMS, the forced swim test (FST) was conducted in all rats after 4 weeks, as previously described.^19^ Preswim exposure was performed 24 h before the test session. During the latter, the total time in seconds spent by the rat without mobility during the 6 minutes was determined. The immobility was considered as “the cessation of limb movement, except for the minor movement necessary to keep the rat aﬂoat”.

The elevated plus maze (EPM) test was performed as previously described.^20^ The number of closed arm entries during 6 min and time spent by each mouse inside the open and closed arms were recorded in seconds.

### Assessment of serum corticosterone, TNF-α, and IL-6 levels

Blood samples were obtained for biochemical assessment from the intra-orbital sinus, centrifuged at 3000 rpm for 15 min at 4°C to obtain the serum then kept at −18°C,

Corticosterone level was measured using ELISA kits (ALPCO Diagnostics, Orangeburg, NY, USA) according to the manufacturers’ instructions. TNF-α, IL-6 (quantakin R&D system, USA Kit) were measured in the serum by ELISA according to the manufacturer instructions.

### Assessment of malonaldehyde (MAD) in the serum

Malonaldehyde (Biodiagnostic; Egypt) was measured for the estimation of damage by reactive oxygen species (ROS) according to the method previously described.^21^

### Assessment of superoxide dismutase (SOD), glutathione peroxidase (GPX), catalase (CAT) in the serum

To assess the antioxidant effect of PE, the levels of SOD (Biodiagnostic; Egypt), GPX (Randox Labs, Crumlin, UK) and CAT (Biodiagnostic; Egypt) were measured in serum according to the methods previously described.^21^

### Quantitative real-time PCR (qRT-PCR)

Ribonucleic acid (RNA) was extracted from 100 mg of formalin-fixed paraffin-embedded (FFPE) section, 1 mL of xylene was used for deparaffinization, incubation at 56°C for 15 minutes, centrifugation for 10 minutes at 13,000 g. The supernatant was discarded, the pellet was washed twice with 1 mL 100% ethanol centrifuged and 1 mL Trizol was added to the pellet.^22^ Extraction of total RNA using Trizol was done according to the supplier instruction (Invitrogen Life Technologies, Carlsbad, CA, USA). The details of the procedure was previously described.^23^

The cDNAs obtained were amplified using PCR Master Mix (Bioneer) with primers designed by Metabion International (Semmelweisser, Germany) as follows: BDNF (forward 5′-TATTTCATACTTCGGTTGC-3; reverse 5′-TGTCAGCCAGTGATGTCG-3′) and β-actin (forward 5′-TCTGGCACCACA CCTTCTA-3; reverse 5′-AGGCATACAGGGACAGCAC-3).

The assay was performed according to a previous study.^22^ The PCR reactions were kept track of by determining the strength of the fluorescence brought on by SYBR Green Dye intercalation to the double-stranded DNA (dsDNA), melting curve evaluation was done to verify the specificity of the products.

### Histopathological assessment

At the end of the experiment, rats were anesthetized with 4% Isoflurane (SEDICO Pharmaceuticals Company, Cairo, Egypt) in 100% oxygen then euthanized by cervical dislocation. Salivary glands were dissected out, fixed in 10% neutral buffered formalin then processed into paraffin blocks to be sectioned at 4-μm thickness and stained with Haematoxylin and Eosin (H&E).

A set of slides were immunohistochemically stained using the streptavidin–biotin–peroxidase technique. Anti-alpha Smooth muscle actin (ASMA) antibody (Biocare Medical, Pacheco, USA, at 1/100 dilution), a marker of myoepithelial cells (MECs).^24^ Anticaspase-3 (Dako Company, Cairo, Egypt, at 1/200 dilution), a marker of apoptosis was used. Anti-BDNF antibody (Santa Cruz Biotechnology, Texas, USA at 1/400 dilution) was also used.

Olympus BX-51 (Tokyo, Japan) microscope connected to digital camera was used to for photographing. Immunoexpression of the studied antibodies (expressed as percentage area) was assessed in 30 fields at ×200 magnification using Pro Plus image analysis software version 6.0. Histopathological assessment was performed by a histopathologist blinded to the experimental groups. The principal investigator was aware of the group allocation at the different stages of the experiment.

### Statistical analysis

Statistical Package for the Social Sciences (SPSS, v.16) was utilized to analyze the data and the results were presented as mean and standard deviations (SD). Data normality was tested using Kolmogorov-Smirnov test. For the parametric data, the different groups were compared using one-way ANOVA (F test), followed by Bonferroni *post-hoc* test. For nonparametric data (e.g., immunohistochemistry and gene expression variables), one-way analysis of variance (ANOVA) (Kruskal-Wallis test) followed by a Dunn’s *post-hoc* test was done to avoid a multiple-comparison effect. The sample size was determined using power analysis. The experimental unit of the study was the rat. No inclusion or exclusion criteria were adopted regarding the animals. No experimental units were excluded during the analysis. Significance was considered at a p<0.05.

## Results

### Analysis of the pumpkin extract

The constituents of PE were determined using GC-MS and were found to include mainly Oleic acid (about 56%), Hexadecanoic acid (about 9%), Decenoic acid (5%) and other components, whereas the Linoleic acid represent only 1% (Table 1).

**Table 1:** Effect of pumpkin extract (PE) on the studied variables

Parameter (in the serum)	Control	CUMS	CUMS+FLU	CUMS+Pump	F/Chi-Square
	(n=10)	(n=10)	(n=10)	(n=10)	
Total immobility time of FST	302.16±7.12	357.25±35.27 p˂0.001	328.11±11.64 p#=0.01	321.16±6.92 p#=0.001	F=14.4
Time spent in the open arm of EPM	27.40±3.51	11.78±0.93 p˂0.001	16.76±2.53 p#˂0.001	17.60±2.18 p#˂0.001	F=70.17
Number of closed arm entries of EPM	17.40±2.59	25.40±1.42 p˂0.001	19.00±4.83 p#=0.001	20.10±3.69 p#=0.01	F=10.50
Serum corticosterone level (ng/mL)	5.67±1.25	11.28±1.80 p˂0.001	5.01±0.42 p#˂0.001	5.19±0.85 p#˂0.001	F=63.11
TNF-α (pg/ml)	29.58±7.84	97.17±11.59 p˂0.001	52.39±11.72 p#˂0.001	42.74±7.69 p#˂0.001	F=87.69
IL-6 (pg/ml)	25.97±3.86	111.82±11.7 p˂0.001	64.39±11.85 p#˂0.001	35.39±6.45 p#˂0.001	F=178.14
MDA (nmoL/ml)	1.35±0.14	2.24±0.71 p˂0.001	1.59±0.36 p#=0.01	1.52±0.18 p#=0.003	F=8.73
SOD (µ/ml)	18.91±2.92	9.945±2.10 p˂0.001	16.48±4.62 p#=0.001	14.99±3.58 p#=0.01	F=12.16
GPX (µ/ml)	58.60±7.76	37.49±4.96 p˂0.001	48.39±10.57 p#=0.02	47.92±6.89 p#=0.03	F=12.19
CAT (µ/L)	0.41±0.09	0.25±0.08 p=0.01	0.38±0.12 p#=0.03	0.38±0.09 p#=0.03	F=5.38
BDNF gene expression in SMG	1.44±0.37	5.86±2.10 p=0.04	10.05±2.12 p#=0.04	12.18±6.03 p#=0.001	Chi-Square= 28.84
BDNF gene expression in Parotid	1.57±0.46	5.39±2.11 p=0.001	8.16±3.17 p#=0.04	8.66±1.97 p#=0.01	Chi-Square= 26.61
BDNF gene expression in SLG	1.73±0.35	5.46±2.57 p˂0.001	8.66±1.97 p#=0.002	8.55±1.49 p#=0.003	Chi-Square= 26.14
Caspase-3 immunoexpression in SMG (%)	4.20±1.32	11.02±2.86 p˂0.001	7.18±2.11 p#=0.02	6.15±1.56 p#=0.002	Chi-Square= 23.50
Caspase-3 immunoexpression in SLG (%)	3.62±1.36	9.21±2 p˂0.001	6.22±2.33 p#=0.03	5.56±2.09 p#=0.01	Chi-Square= 20.51
Caspase-3 immunoexpression in Parotid (%)	3.81±1.19	9.43±3.11 p=0.001	5.37±2.31 p#=0.04	5.31±1.40 p#=0.01	Chi-Square= 19.89
ASMA immunoexpression in SMG (%)	10.42±2.92	4.00±1.56 p˂0.001	7.30±2.00 p#=0.004	8.10±2.68 p#=0.01	Chi-Square= 20.44
ASMA immunoexpression in SLG (%)	5.01±2.04	1.64±0.62 p#=0.003	4.12±1.97 p#=0.02	3.69±1.59 p#=0.02	Chi-Square= 18.09
ASMA immunoexpression in Parotid (%)	3.89±1.08	1.74±0.65 p˂0.001	3.17±1.33 p#=0.04	3.41±1.53 p#=0.04	Chi-Square= 15.41
BDNF immunoexpression in SMG (%)	9.97±2.56	15.87±3.31 p=0.002	21.95±3.75 p#=0.01	25.04±7.10 p#=0.02	Chi-Square= 25.80
BDNF immunoexpression in SLG (%)	4.24±1.29	10.89±3.67 p=0.001	17.45±4.68 p#=0.02	20.18±7.47 p#=0.02	Chi-Square= 28.46
BDNF immunoexpression in Parotid (%)	3.81±1.19	11.04±3.32 p˂0.001	16.66±4.72 p#=0.04	19.77±8.03 p#=0.04	Chi-Square= 26.64

The data were compared using one-way ANOVA test, followed by Bonferroni post-hoc test and one-way ANOVA (Kruskal-Wallis test) followed by a Dunn’s post-hoc test, (n=10). Data are shown as mean ± SD.

P represents p value versus control group.

P# represents p value versus CUMS group.

CUMS: chronic unpredictable mild stress; FLU: Fluoxetine; Pump: pumpkin; ASMA: alpha-smooth muscle actin; BDNF: brain-derived neurotropic factor; SMG: submandibular gland.

### Effect of exposure to CUMS on corticosterone

**Figure 1:**
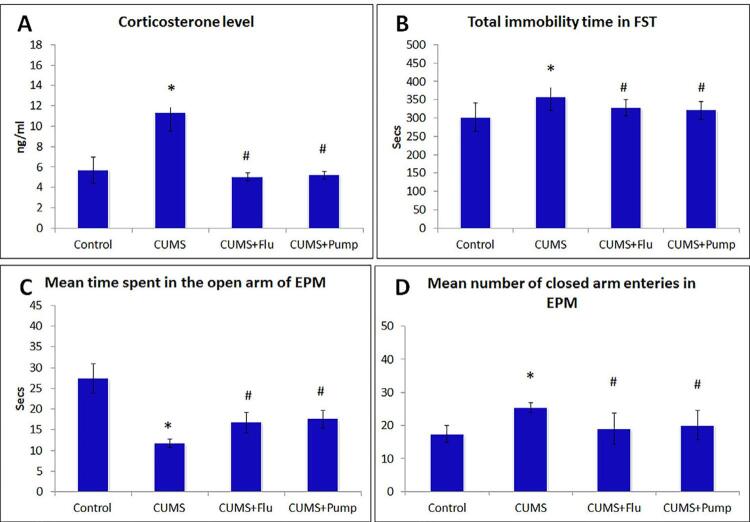
Confirmation of the depressive status in rats after exposure to CUMS was done through assessing serum corticosterone level (A), immobility time (B) during forced swimming test (FST) and elevated plus maze test (EPM) (C, D). CUMS: chronic unpredictable mild stress, FLU: fluoxetine, Pump: pumpkin. Data were compared using one way ANOVA test, followed by Bonferroni post-hoc test, (n=10). * indicates significant difference compared to the control group, # indicates significant difference compared to the CUMS group. Significance is considered at p˂0.05

After 4 weeks of exposure to CUMS, a significant (p<0.001) increase in the serum corticosterone level in CUMS-exposed rats was observed. Both FLU (p=0.002) and Pump (p<0.001) administration significantly reduced serum corticosterone level (Figure 1A, Table 1).

### Effect of exposure to CUMS on behavior

Exposure to CUMS leads to development of depressive-like behavior that was evident in the rat by the significant increase (p<0.001) in the mean immobility time compared to the control. Administration of FLU and Pump along with the CUMS reduced the depressive-like behavior, as evidenced by the significant decrease (p=0.02, p<0.001) in immobility time compared to CUMS, respectively (Table 1 and Figure 1B).

This was also confirmed by the EPM test that revealed a significant decrease (p<0.001) in the time spent by rats of CUMS group in the open arm compared to the control group. Administration of FLU and Pump significantly increased (p<0.001) the time spent in the open arm compared to the CUMS group. Also, the number of closed arm entries was significantly increased (p<0.001) after CUMS compared to the control group, whereas administration of FLU (p<0.001) and Pump (p=0.001) significantly decreased it compared to the CUMS group (Table 1 and Figure 1C, 1D).

### Effect of PE on the anti-inflammatory cytokines

**Figure 2:**
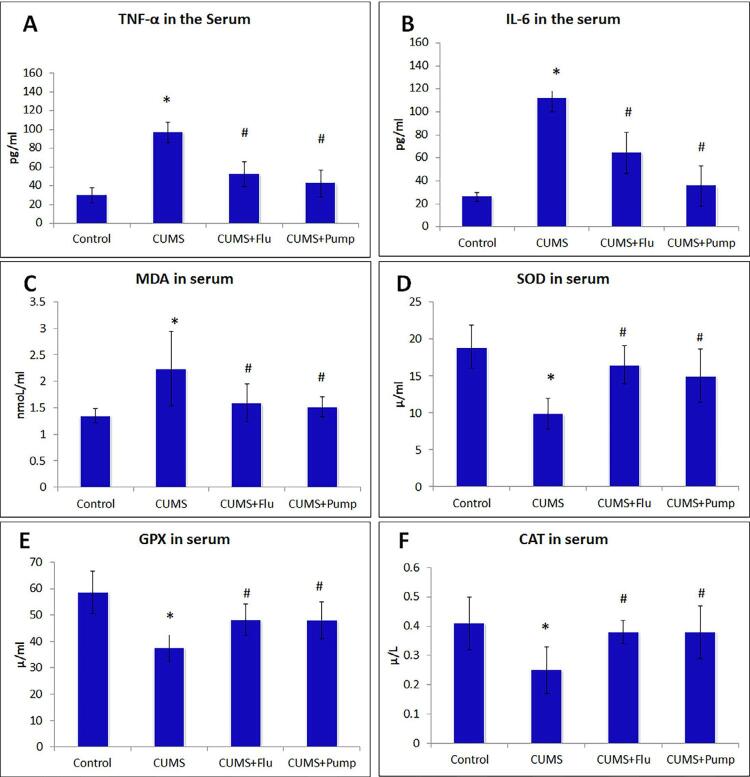
Effect of Pumpkin extract on the pro-inflammatory cytokines TNF-α (A) and IL-6 (B), oxidants (MDA) (C) and antioxidants; SOD (D), GPX (E) and CAT(F) levels in the serum of the studied groups. Data were compared using one way ANOVA test, followed by Bonferroni post-hoc test, (n=10). Results are shown as mean ± SD. * indicates significant difference compared to the control group, # indicates significant difference compared to the CUMS group. Significance is considered at p˂0.05

The pro-inflammatory cytokines levels were measured to detect the anti-inflammatory effect of PE. Serum TNF-α, IL-6 levels were significantly increased (p<0.001) in CUMS group compared to the control, whereas their levels significantly decreased (p<0.001) in both FLU and Pump-treated groups (Figure 2A, 2B and Table 1).

CUMS: chronic unpredictable mild stress, FLU: fluoxetine, Pump: pumpkin. (TNF-α (tumor necrosis factor-α), interleukin-6 (IL-6), SOD (superoxide Dismutase), MDA (Malondialdehyde) GPX (Glutathione peroxidase), and CAT (catalase) in the serum

### Effect of PE on the oxidant/antioxidant profile

**Figure 3:**
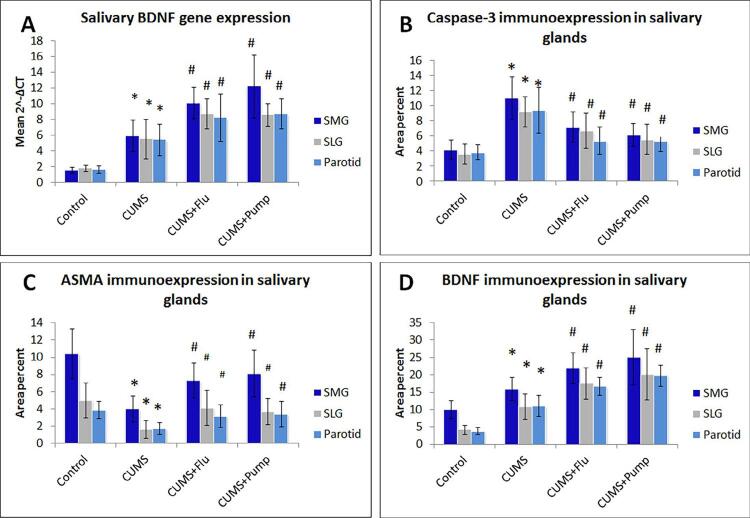
Effect of Pumpkin extract on salivary gene expression of BDNF using qRT-PCR, immunoexpression of caspase-3, BDNF.Data were compared using one way ANOVA (Kruskal-Wallis test) followed by a Dunn’s post-hoc test, (n=10). Data are shown as mean ± SD. * indicates significant difference compared to the control group, # indicates significant difference compared to the CUMS group. Significance is considered at p˂0.05

Exposure to CUMS was associated with a significant increase (p<0.001) in serum MDA, whereas it significantly decreased (p=0.03) in Pump-treated group among the treated groups (Figure 3C).

CUMS: chronic unpredictable mild stress, FLU: fluoxetine, Pump: pumpkin. ASMA: alpha-smooth muscle actin, BDNF: brain-derived neurotropic factor, SMG: submandibular gland

Exposure to CUMS was also associated with a significant decrease in the serum SOD (p<0.001), GPX (p<0.001) and CAT (p=0.001) compared to the control group. On the other hand, Pump-treated group showed a significant increase (p=0.004, p=0.003, p=0.02) in the serum levels of SOD, GPX and CAT, respectively (Figure 3D-F).

### Effect of PE on salivary gene expression of BDNF

Gene expression of BDNF was measured in the salivary glands of the studied groups. The level of BDNF transcription significantly increased (p<0.001) in CUMS compared to the control, as in both FLU (p<0.001) and Pump-treated (p<0.001) groups compared to the untreated CUMS group (Figure 3A).

### Effect of PE on the histological structure of the salivary glands

#### Submandibular gland (SMG)

**Figure 4:**
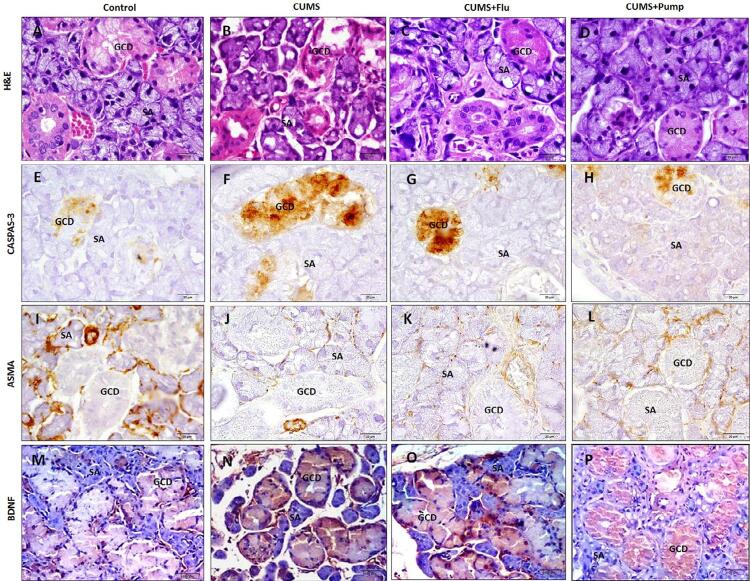
Sections of the SMG of control group show intact seromucous acini (SA) and numerous large granular convoluted ducts (GCD). Degenerative changes and vacuolation of some SA and GCD appear in the SMG of the CUMS group while those of CUMS+FLU and CUMS+Pump groups show SA with few vacuolations and intact GCD (H&E (A-D)X 400). Immunoexpression of Caspase-3 (E-H), ASMA (I-L) and BDNF (M-P) in the studied groups are represented (immunostaining ×400). Scale bars = 20 µm

In control rats the SMG was formed of intact seromucous acini, numerous well developed large granular convoluted ducts (GCD) and other ductal components. Exposure to CUMS resulted in degenerative changes and vacuolation in some seromucous acinar cells and GCD. Treatment with FLU or Pump slightly decreased the CUMS-induced vacuolation in acinar cells with restoration of GCD structure (Figure 4A-D).

CUMS: chronic unpredictable mild stress, FLU: fluoxetine, Pump: pumpkin, SMG: submandibular gland, ASMA: alpha smooth muscle actin, EGF: epidermal growth factor, GCT; granular convoluted ducts, BDNF: brain-derived neurotropic factor)

Immunohistochemical staining with caspase-3 was done to detect the cellular apoptosis. The SMG of control group showed minimal caspase-3 immunoreactivity in the acini and duct cells, whereas exposure to CUMS lead to significant increase (p<0.001) in caspase-3 expression compared to the control rats. Whereas treatment with FLU or Pump significantly decreased the expression of caspase-3 (p=0.03, p=0.01) compared to the untreated rats (Figure 3B, Figure 4E-H).

Immunostaining with ASMA was performed to assess the integrity of the myoepithelial cells (MECs) present around the glandular acini and ducts. We observed that SMG of the control group had strongly positive immunoreactivity for ASMA, whereas exposure to CUMS resulted in a significant reduction (p<0.001) in this reaction compared to the control. There was a significantly increase (p<0.001) of ASMA expression in both salivary acini and ducts in CUMS+Pump group compared to the CUMS group, whereas no such effect was detected in CUMS+FLU group (Figure 3C, Figure 4I-L). BDNF was weakly expressed mainly in the duct system of SMG of the control group, whereas was significantly increased (p<0.001) after exposure to CUMS compared to the control rats. Further significant increase (p=0.03, p=0.01) of BDNF was observed in FLU- or Pump-treated groups when compared to the CUMS group (Figure 3D, Figure 4M-P).

#### Parotid gland

**Figure 5:**
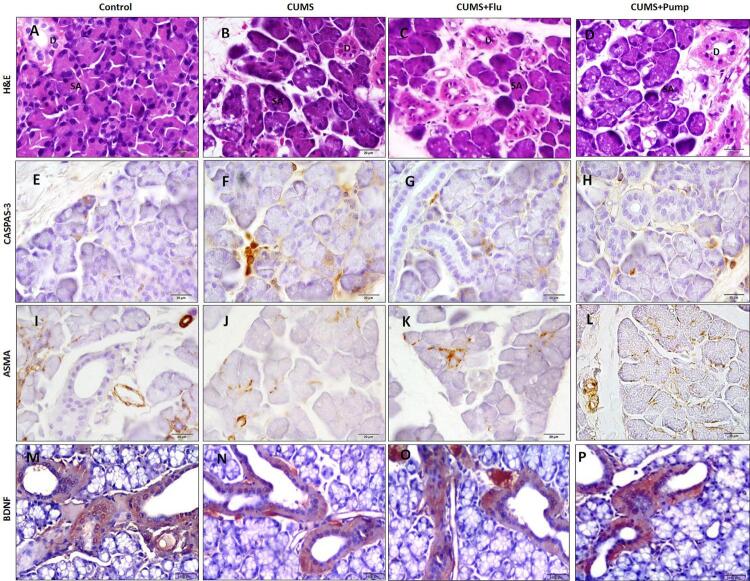
Sections of the parotid gland of control group show intact serous acini (SA) and ducts (D). Gland of CUMS group show atrophied acini lined by dark nuclei with vacuolated cytoplasm and pyknotic nuclei and those of CUMS+FLU and CUMS+Pump groups show fewer atrophic acini while their acini still have vacuolated cytoplasm (H&E (A-D)×400). Immunoreactivity for caspase-3(E-H), ASMA (I-L) and BDNF M-P) in the studied groups are represented (Immunostaining ×400). Scale bars = 20 µm

The parotid gland of control rats was formed of closely packed, intact pure serous acini and striated ducts, besides the other ductal components. After exposure to CUMS, the serous acini appeared smaller in size and some of them were atrophic, whereas some of the acinar cells were vacuolated. These changes were also observed in the glands of rats treated with FLU or Pump, but to a lesser degree (Figure 5A-D and Table 1).

CUMS: chronic unpredictable mild stress, FLU: fluoxetine, Pump: pumpkin, ASMA: alpha smooth muscle actin, BDNF: brain-derived neurotropic factor.

Parotid glands of control group showed minimal caspase-3 immunoreactivity, whereas it significantly increased (p<0.001) after exposure to CUMS compared to the control rats. Both FLU and Pump-treated groups showed significantly increased (p=0.03, p=0.01) caspase-3 expression compared to the CUMS group (Figure 3B, Figure 5E-H). A significant decrease (p<0.001) in ASMA immunoreactivity was observed in the parotid gland CUMS group. Although treatment with FLU increased the expression of ASMA, this increase was not statistically significant, whereas treatment with Pump significantly increase (p<0.001) compared to CUMS group (Figure 3C, Figure 5I-L).

Immunoreactivity for BDNF was detected in striated ducts of the control parotid glands, which was significantly increased (p<0.001) following the exposure to CUMS compared to the control group, and further significantly increased (p<0.04, p=0.02) in FLU and Pump-treated groups compared to the CUMS group (Figure 3D, Figure 5M-P).

#### Sublingual gland (SLG)

**Figure 6:**
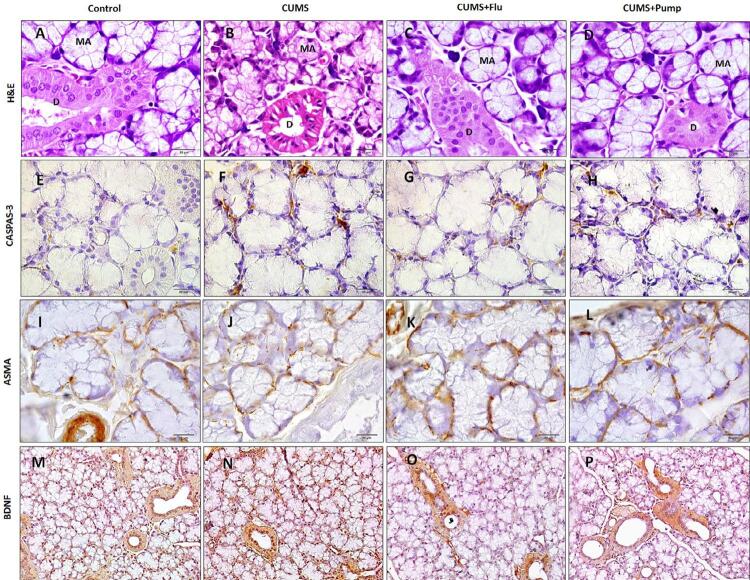
Sections of the SLG of the control show intact mucous acini (MA) and ducts (D). SLG of CUMS group show many atrophied MA and ducts with vacuolated cells while most of them in FLU- or Pump-treated appear intact (H&E (A-D)×400). Immunoreactivity for caspase-3 (E-H), ASMA (I-L) and BDNF (M-P) the studied groups are represented (Immunostaining ×400). Scale bars = 20 µm

We observed that the SLG of the control group was formed of closely packed, intact pure mucous acini (MA), striated ducts and other ductal components. After exposure to CUMS, many MA and ducts appeared smaller with disorganized architecture and vacuolated cells. Most of the acini and ducts of SLG in FLU or Pump-treated appeared intact (Figure 6 A-D).

CUMS: chronic unpredictable mild stress, FLU: fluoxetine, Pump: pumpkin, ASMA: alpha smooth muscle actin, BDNF: brain-derived neurotropic factor.

Expression of caspase-3 significantly increased (p<0.001) after exposure to CUMS, whereas the FLU and Pump-treated significantly decreased (p=0.04, p=0.001) compared to the CUMS group (Figure 3B, 6E-H). A significant decrease (p<0.001) of ASMA immunoreactivity was detected in SLG of CUMS group, whereas treatment with Pump significantly increased (p<0.001) it compared to the CUMS group (Figure 3C, Figure 6I-L).

Immunoreactivity for BDNF was detected in striated ducts of the SLG. It significantly increased (p<0.001) after exposure to CUMS and further increased after administration of FLU or Pump (p<0.04, p=0.02) compared to the CUMS (Figure 3D, Figure 6M-P).

## Discussion

Increase incidence of depression requires serious attention by health care providers.^25^ Many methodological and ethical questions constrain the exploration of dynamic changes during depression in humans. Therefore, creating suitable animal models of depressive-like status has become a standard research technique for this purpose.^26^ First, we performed the most suitable depressive-like model of CUMS, a stress rat and mouse model, in which the reward reflex activity is impaired.^27^

Saliva is essential to preserve oral health, since alterations in the salivary gland function could affect oral tissue integrity.^28^ Function of the salivary glands is essentially controlled by the autonomic nervous system, so it is directly affected on exposure to stresses.^28^ BDNF is among the stress markers in saliva that have been identified in previous studies. We described BDNF measurement as a useful tool in evaluating the activation of the sympathetic adrenomedullary system.^29^ This is the reason why our study focused on BDNF.

Our study aimed to investigate the effect of exposure to CUMS on the structure of rat salivary glands structure and the possible effect of PE in ameliorating this impact. We noticed that exposing rats to CUMS for 4 weeks resulted in the development of a depressive status and anxiety evidenced by the prolongation of the immobility time of the FST and by the EPM test, respectively. An increase in the amount of serum corticosterone was correlated with this depressive-like state. These results were in line with some previous studies.^9,30^

The observed significant increase of serum corticosterone level, known as a stress marker, in the chronically stressed rats was due to the overactivity of hypothalamus–pituitary–adrenal (HPA) system mediating the reaction to stress.^31^ The levels of salivary cortisol are strongly associated with the levels of blood cortisol and represent HPA activity reliably. Different sources of psychological and social stress were behind the stimulation of the HPA system and led to significant rise in salivary cortisol levels.^32^

The neurobiology of depression was known from the unexpected discovery of the antidepressant activities of drugs which augment the neurotransmission of brain monoamines.^33^ In our study, both FLU and Pump administration significantly ameliorated the depressive-like status induced by CUMS. Studies have described the antidepressant-like effect of pumpkin,^34^ reporting that oral SSP markedly decrease the immobility time in FST and increase the levels of BDNF. This knowledge motivated us to choose pumpkin to relieve depressive-like status.

In our study, exposure to CUMS induced degenerative changes in cells of the secretory and ductal system, evident by the presence of vacuolation along with atrophy of acini of the glands and increased apoptosis of the acinar and ductal cells. Furthermore, the structural integrity of acinar and ductal MECs was affected by CUMS exposure, evident immunohistochemically using ASMA antibodies, which subsequently disturbed their contractility and decreased salivary secretion. The increased level of serum corticosterone could be the cause of stress-induced histopathological changes in the salivary glands, such as the similar changes previously recorded in the submaxillary glands of rats following injection of hydrocortisone for 14 days.^35^

Malondialdehyde is a lipid peroxidation product causing oxidation of polyunsaturated fatty acids, and leading to oxidative stress and generation of highly reactive oxygen species (ROS) directly involved in oxidative damage of cellular macromolecules.^36^ Stress and high glucocorticoids level were described to cause excitotoxicity of glutamate, disrupt homeostasis of calcium, hinder the transport of glucose and increase the production of oxygen radicals.^6^ In our study, exposure to CUMS was associated with a significant increase in serum MDA, which indicates the role of oxidative stress in the induction of stress-induced depressive-like status. These findings were consistent with those previously conducted on experimental chronic stress.^37^

Antioxidants act as “free radical scavengers” that prevent and recover the harmful effects of ROS. CAT is the main antioxidant enzyme implicated in hydrogen peroxide (H_2_O_2_) degradation.^38^ In our study, exposure to CUMS was associated with a significant reduction in serum SOD, GPX and CAT which indicates decreased total antioxidant capacity. Moreover, many studies showed that CUMS procedure markedly resulted in an oxidative damage in the prefrontal cortex, striatum, and hippocampus of rats, evident by up-regulation of MDA and down-regulation of SOD and CAT.^39^ The previous data support our suggestions that oxidative stress, evident in our study, could be also behind the pathological alterations noted in CUMS-exposed salivary gland. Therefore, the structural observations were strengthened and explained by the biochemical results.

Pump-treated rats showed a notable elevation in antioxidant enzymes SOD, GPX and CAT in the serum indicating increased total antioxidant capacity. Several studies have evidenced the antioxidant capacity of pumpkin in different tissues,^40^ which justified its use in our study to alleviate the oxidative stress associated with depressive-like status. Chronic antidepressant treatments were reported to ameliorate oxidative damage in the different brain areas involved in mood control in rat.^18^ Oleic acid (56%) represents the main constituents of pumpkin fruit extract, used in our study, whereas Linoleic acid represents only about 1%. Studies have already reported a potent antioxidant effect of these components, and our findings partially agreed with those of Bora, et al.^41^(2019).

Since inflammation has been proposed to be a causative mechanism in the development of chronic stress-induced depression,^7^ we assessed the inflammatory cytokines TNF-α and IL-6. The results showed a notable elevation of serum TNF-α and IL-٦ of CUMS-exposed rats indicating the inflammatory process associated with chronic stress-induced depressive-like status. This is in agreement with previous studies that described depression as an oxidative and inflammatory disorder.^8^ Furthermore, inflammatory cytokines, IL-1 β, IL-6 and TNF-α, reported to be elevated in rats that exhibited a depressive-like behavior.^42^ These cytokines interfere with the metabolism of serotonin, an important neurotransmitter, and interfere with the synaptic plasticity by altering of BDNF and its receptor, tropomyosin receptor kinase B (TrkB) that plays a crucial role in the pathophysiology of depression in the prefrontal cortex and hippocampus.^43^ In our study, FLU and Pump administration markedly reduced serum pro-inflammatory cytokines. These findings were consistent with those of Kim, et al.^34^ (2016), who reported that pumpkin significantly reduced the protein levels of TNF-α and IL-6 in the serum of rats that exhibited a depressive-like behavior animals.

In our study, immunohistochemical detection of BDNF in the ductal cells confirmed that salivary glands produced BDNF protein. Immunoexpression of BDNF in SMG, SLG and parotid glands notably up-regulated on exposure to CUMS. This was confirmed by the increase in BDNF mRNA in the salivary glands. These findings also reported, by some researchers, an increase in salivary gland expression of BDNF after exposure to different types of stress.^9,28^ This indicated that salivary gland is sensitive and responsive to stress as evidenced by increased BDNF expression.

In our study, treating rats from depressive-like status resulted from exposure to CUMS with fluoxetine or pumpkin induced a notable up-regulation of salivary BDNF expression compared to the untreated rats. These findings are supported by previous studies that showed that FLU and Pump increased hippocampal BDNF level in CUMS-exposed mice.^6,34^ Saruta, Sato and Tsukinoki^6^ (2010) stated that “secretion of BDNF may represent a protective mechanism that plays important roles in homeostasis under stress conditions”. Therefore, the structural, biochemical and molecular observations collected in this study, suggested the BDNF as a molecular mechanism through which Pumpkin could modulate the protective effect on the salivary glands during chronic stress.

In conclusion, our study showed that Pump ameliorates depressive-like status resulted from exposure to chronic stress by exerting a promising antioxidant, anti-inflammatory and antidepressant-like effects. Pump subsequently improves the structural changes induced by stress in the salivary glands which might be due to up-regulation of salivary BDNF expression. We also recommend future studies to test the efficacy of pumpkin in improving stress-related salivary alterations in humans.
